# Pit-bull reviewing, the pursuit of perfection and the victims of success

**DOI:** 10.1186/1741-7007-9-84

**Published:** 2011-12-01

**Authors:** Miranda Robertson

**Affiliations:** 1 BMC Biology, BioMed Central Ltd, Floor 6, 236 Gray's Inn Road, London, WC1X 8HB, UK

## Editorial

Earlier this year, Hidde Ploegh published in *Nature* a *cri de coeur* on the tyranny (sic) of reviewers [1]; more recently, Gregory Petsko, not (according to his own account [2]) generally an advocate of the addition of yet more journals to the existing myriad, extended a welcome [3] to the news of the open access publication to be launched next year by the Howard Hughes Institute, the Wellcome Trust and the Max Planck Institute. Last month, HHMI, Wellcome and MPI released, along with the name of the new journal – *eLife* – a short manifesto in which they explain what the journal is intended to achieve, and why, and how it will be done.

Meanwhile, our most viewed article for the past year has been Virginia Walbot's 'Are we training pit bulls to review our manuscripts?' [4].

Ploegh, Petsko and Walbot have, with considerable eloquence and varying degrees of passion, described the problem that *eLife* is intended to address: the success of a postdoctoral Fellow in finding a good academic position is perceived to depend, and to a large extent probably does depend, on his or her having published a paper in one of the three highest-profile general biology journals; but getting a paper into one of those journals can be extraordinarily difficult because – it is widely felt (and see [1-3]) – referees seem to see it as their responsibility to insist on time-consuming additions and revisions, and editors are unable or unwilling to judge for themselves the justice of the referees' advice.

Virginia Walbot [4] has suggested how the reviewer problem could be avoided by training graduate students to adopt a more constructive and judicious approach to refereeing. We have for the past three years or so been operating a policy of 're-review opt-out' [5]: authors who have been asked to make substantive revisions to their papers are also asked whether they wish the referees to see the revised version; if not, the decision is made by the editors (more below on how this policy has worked in practice).

The solution proposed by *eLife* is to ensure quality, speed and justice by deploying a high-powered editorial board who will oversee the reviewing process and 150 highly selected biomedical experts who will do the reviewing; to avoid iterations by making a yes-or-no decision on first review; and to promote fairness and transparency by publishing the (anonymous) referees’ reports. This is not very different in principle from the way that at least some other general biology journals operate; and the stated aims of eLife – to deliver quick, fair and intelligent (‘high-quality’) decisions – are, I imagine, shared by all journals aspiring to the selective publication of papers with claims to general interest.

Why are these aims not (apparently) being met by the journals in which postdocs feel they must publish?

The belief that has driven the development of *eLife* is that it should be professional scientists who make decisions on publication, and the perceived problems arise when they are made by professional editors. There are obvious reasons (rehearsed, for example, by Petsko  [3]) to expect that professional scientists will make better decisions than professional editors on scientific papers. But this raises the question of why and how the three journals that are currently perceived to have a stranglehold on the careers of young biologists, all of them run by professional editors, came to be in that position.

Clearly I am an interested party in this argument, so before presenting a few points bearing on the issues, I should like to state that I think a perfectly good case can be made both for professional editors and for professional scientists as the ultimate adjudicators on scientific submissions, and it is a good idea to have journals operating on both systems. I should also add the disclaimer that I am sure there is nothing in what I have to say that the funders and extremely distinguished professionals at present engaged in launching *eLife* are unaware of.

## Principle and practice

It is clear that scientists will be better equipped to evaluate scientific papers, and indeed one anothers’ evaluations of scientific papers, than professional editors with a scientific background. But to meet the aspirations of *eLife* (or any other would-be general-interest selective biology journal) demands a breadth and depth of knowledge, with the sagacity to apply it appropriately, that are in limited supply, as is the time of professional scientists, especially knowledgeable and sagacious ones.

The main arguments in favor of professional editors are that they can make a full-time commitment to their editorial role, and they are less likely to be influenced by personal scientific prejudices and history (their own and those of close colleagues) than are field scientists. Their effectiveness however depends entirely on their willingness to draw on the scientific community for expert advice, not only as referees, but also for perspective on difficult decisions and adjudication in cases of conflict or dispute. Woe betide the professional editor who thinks she or he can judge the issues without reference to the real experts.

Benjamin Lewin has long since retired from *Cell*, so it is not, I hope, invidious to cite him as an example of a professional editor (he was also of course the publisher) with the vision and understanding to launch what was and is now by any criterion an egregiously successful and high-quality journal. (Lewin – and Cell – have some distinguished detractors, as well as many admirers; but you don’t get distinguished detractors without having achieved something important. Perhaps here I should follow Petsko’s example [3] of full disclosure, and volunteer that Lewin and I were for a while colleagues on the editorial staff of *Nature*, where we fought like cat and dog.)

Of course, some professional editors are better than others. So are some scientists, in an editorial capacity. The main difference is that research scientists constitute a community within which the quality of an individual can reasonably reliably be judged by consensus criteria, whereas no such community or consensus criteria exist for editors.

However, it seems to me that the problem that is actually at the root of the frustrations with publishing in the biomedical sciences is the scarcity of expertise, breadth and sagacity relative to the number of papers on which their authors have a right to expect they will be exercised. This simply makes it very difficult to achieve consistently fair and intelligent decisions, and it is even more difficult to couple this with speed.

Worse, once a journal has become one of the high-profile, high-impact-factor few in which all ambitious postdocs hope to publish, there is a danger not only that the number of submissions will exceed any reasonable capacity for consistently making good quick decisions, but that the incentive to editors to ensure such decisions concomitantly decreases, since the reputation of the journal is by that time sufficient to ensure its continued success in the absence of anything but egregious and consistent lapses. (And possibly even then.)

In this way, aspiring young authors can become the victims of the journals’ success.

I don’t think there is an infallible formula for getting the publishing process right. But there are certainly useful guidelines. Apropos of which, I promised to return to our own experiment in the pursuit of perfection in the editorial process.

## Re-review opt-out and the pursuit of perfection

When we initiated the policy of allowing authors to choose whether their revised manuscripts were subjected to a second scrutiny by their referees, our main fear was not the risk of publishing invalid science, but that of losing good referees. This fear proved unfounded (although there is no way of knowing whether this is simply because most referees never read far enough down the invitation letter to reach the paragraph explaining that the authors may opt out of re-review).

How have authors responded? Roughly half of all authors choose not to go back to referees after revising their papers. As editors, if we are in doubt about the validity of the revised paper, we generally reject it. In that case, authors sometimes appeal and ask us to consult the referees again, and on one or two occasions, the referees have proved more tolerant than we were (usually this is to do with the level of validation that should reasonably be required).

Often, authors would prefer on balance to have their revised manuscripts ratified by the reviewers, but want to avoid delay: in that case, we generally offer to consult referees again with the proviso that if they don’t respond within a week we will make a decision without them.

In the interests of avoiding delay to the publication of papers at least some of whose results are valid and worthy of publication, even if they do not quite meet the strongest claims of the authors, we also often offer authors the alternatives of strengthening their paper for possible publication in BMC Biology, or resubmitting to one of our subject-specific sister journals of the BMC series where it may be acceptable with only minor or no revisions to meet the existing referees’ criticisms. We consider this an extremely important service to authors submitting to a journal that maintains a threshold based on a judgement of the importance or general interest of the papers submitted to it, and not just their scientific soundness.

Do we, in consequence of our policy, make more mistakes than other journals? It’s much too soon to say.

## Footnotes

Finally, I should like to return to more general issues, and to endorse strongly one remark of Hidde Ploegh [1], and to challenge another made by our valued friend, advisor and Editorial Board member Gregory Petsko [3]

It is very important, as Ploegh recommends [1], that editors be willing to take the time to establish, by consulting appropriate experts, how important and how reasonable the demands of referees for additional experiments may be; and that they make their position transparent to the authors in delivering their decision and don’t equivocate. (This is not trivial to achieve – *vide supra*.)

Petsko, I think, is a little unfair in castigating the high-profile journals for publishing a relatively high proportion of papers that turn out to be seriously wrong. Journals with a claim to general interest should be prepared to take risks on non-mainstream papers, and they will inevitably sometimes prove wrong. (That said, it should be possible to avoid publishing papers that were clearly wrong, or highly likely to prove wrong, at the time of publication, and it seems this is not always avoided.)

I also have some comments on the issue of length and the absence of page limits; but enough is enough, and they will have to wait for another editorial.
